# Discrepancies between Skinned Single Muscle Fibres and Whole Thigh Muscle Function Characteristics in Young and Elderly Human Subjects

**DOI:** 10.1155/2016/6206959

**Published:** 2016-12-14

**Authors:** Hyunseok Jee, Jae-Young Lim

**Affiliations:** ^1^Department of Rehabilitation Medicine, Seoul National University Bundang Hospital, 300 Gumi-dong, Bundang-gu, Seongnam-si, Gyeonggi-do 463-707, Republic of Korea; ^2^Institute on Aging, Seoul National University, Seoul, Republic of Korea

## Abstract

We aimed to analyse the mechanical properties of skinned single muscle fibres derived from the vastus lateralis (VL) muscle in relation to those of the whole intact thigh muscle and to compare any difference between young and older adults. Sixteen young men (29.25 ± 4.65 years), 11 older men (71.45 ± 2.94 years), 11 young women (29.64 ± 4.88 years), and 7 older women (67.29 ± 1.70 years) were recruited. In vivo analyses were performed for mechanical properties such as isokinetic performance, isometric torque, and power. Specific force and maximum shortening velocity (Vo) were measured with single muscle fibres. Sex difference showed greater impact on the functional properties of both the whole muscle (*p* < 0.01) and single muscle fibres than aging (*p* < 0.05). Sex difference, rather than aging, yielded more remarkable differences in gross mechanical properties in the single muscle fibre study in which significant differences between young men and young women were found only in the cross-sectional area and Vo (*p* < 0.05). Age and sex differences reflect the mechanical properties of both single muscle fibres and whole thigh muscle, with the whole muscle yielding more prominent functional properties.

## 1. Introduction

Aging of the human skeletal muscle is characterized by declines in mechanical, morphological, and functional properties such as the cross-sectional area (CSA) of each myocyte, specific force (SF), maximal contractile force, and maximal shortening velocity (Vo) [1–3]. However, other muscle fibre mechanical studies on aging did not find the specific force and Vo of muscle fibres to vary remarkably [4–6].

Furthermore, many studies reported that the aging process causes mechanical changes in the muscle, such as increasing muscle weakness and slowing the contraction speed [2, 7, 8]. However, the differences in the results of these studies are controversial, in terms of the concept of age-related mechanical changes at the single muscle cell level.

Various studies, ranging from the micro- to macrolevels, have investigated the quantitative aspects of muscle changes, such as muscle mass [9]. Gradually impaired skeletal muscle function is caused not only by the quantitative aspect such as progressive reduction in muscle mass (sarcopenia), but also by age-related muscle qualitative aspect [10]. This qualitative aspect in macrolevel is also considered as decreased muscle short-term power and/or optimal shortening velocity by aging [11, 12].

However, minute observations of qualitative changes of muscle function as a result of aging per se are more important, as microscopic changes are usually early signs of whole-body changes [13]. These observations can be attained through microscopic analysis of single muscle fibres.

At the single muscle fibre level, myosin heavy chain (MHC) type II-related fast fibres showed faster decline with aging than did slow MHC type I, demonstrating that MHC II is sensitive to the aging process [2]. However, a shift to type IIx or hybrid type in single muscle fibres was also reported in aging, whereas other studies showed that fast muscles decreased with aging [14]. In a previous study, functional differences according to sex were not found at the single muscle fibre level [13]. Sex or age differences have a more distinct relation with strength at the whole muscle level than at the single muscle fibre level, even within the same subject [6, 15, 16].

To examine the opposite controversial issue regarding single muscle fibres in relation to in vivo whole thigh muscle function in young and older men and women, we hypothesized that (i) there is a difference between young and older adults and between men and women at the single muscle fibre level and (ii) the differences of age and sex at the single muscle fibre level are relatively reflective of those at the whole muscle level.

To ascertain whether sex and/or aging causes differences in mechanical properties at the single muscle fibre and at the whole muscle level, we compared young and older Korean men and women and analysed the mechanical functions of skinned single muscle fibres derived from the VL muscle, in contrast to those of whole thigh muscles.

## 2. Materials and Methods

### 2.1. Subjects' Characteristics

This study was designed to investigate the functional response of single muscle fibres and whole thigh muscle of healthy young men (YM; age 29.25 ± 4.65 years, height 176.84 ± 2.52 cm, and weight 75.28 ± 9.15 kg), healthy older men (OM; 71.45 ± 2.94 years, 164.10 ± 5.18 cm, and 68.24 ± 6.51 kg), healthy young women (YW; 29.64 ± 4.88 years, 160.35 ± 4.57 cm, and 56.79 ± 10.64 kg), and healthy older women (OW; 67.29 ± 1.70 years, 155.69 ± 6.10 cm, and 58.01 ± 9.71 kg) (Table 1). A total of 45 community dwelling young and old adults (16 YM, 11 OM, 11 YW, and 7 OW have not been joining exercise related activities for the recent 3 years) were recruited from healthy volunteers who agreed to participate in this study from February 2014 to April 2015. The healthy adults were defined as (i) scored ≥ 10 on the Short Physical Performance Battery (SPPB); (ii) nondisease with nonmedication; and (iii) nondrinking and nonsmoking after completing a comprehensive medical evaluation which included a history and physical test [17]. The subjects provided written informed consent before the study commencement. The study was approved by the institutional review board at Seoul National University Bundang Hospital (B-1307-212-008).

### 2.2. Isometric, Isokinetic, and Power Performance Test of the Left Thigh in YM, OM, YW, and OW

For the in vivo study on the mechanical properties of thigh muscle function before VL muscle biopsy, we performed isometric, isokinetic, and power performance tests of knee flexion and extension (Primus RS; BTE, Hanover, MD, USA). Subjects were firmly fixed on a seat in the sit position. The speed and range of isokinetic and isometric test are 60°/sec and 65°, respectively. The power value was obtained whilst subjects exhibit maximal power against resistance automatically taken by BTE. The measured parameters were as follows: isometric test of the left thigh, Iso; power test, Po; isokinetic test of the left thigh extension peak torque, IsoKE; and isokinetic test of the left thigh flexion peak torque, IsoKF (Figure 3). All variables measured as peak values are given in newton meter (Nm), except for Po, which is expressed in watts (W).

### 2.3. Muscle Biopsy

Whole participants in this study visited three times (each of visits was at different date) for (i) screening participants at the first visit; (ii) testing physical function at the second visit; and (iii) obtaining muscle specimens at the third visit. Muscle specimens were obtained percutaneously from the VL muscle under local anaesthesia by using a modified Bergstrom needle (11750-06 and 11750-07; Dixons, Wickford, UK). One portion of the tissue was immediately soaked in cold relaxation solution (4°C), as described below; the tissue was subsequently dissected into small bundles of muscle fibres for use in the functional test. The bundles were then stored in 50% glycerol (v/v) solution at −20°C for the subsequent functional study. All bundles for the functional tests were analysed within 2 weeks, which is based on dates of degraded quality in single muscle fibre mechanics (Table 4).

### 2.4. Single Muscle Fibre Solution

The skinned single muscle fibre solution consisted of 40 mM* N*,*N*-bis(2-hydroxyethyl)-2-aminoethanesulphonic acid (BES); 10 mM ethylene glycol tetraacetic acid (EGTA); 6.56 mM magnesium chloride (MgCl_2_); 5.88 mM sodium-adenosine triphosphate (Na-ATP); 46.35 mM potassium- (K-) propionate; 15 mM creatine phosphate; 1 mM dithiothreitol; 10% Triton X-100; and protease inhibitors such as 0.01 mM E64, 0.047 mM leupeptin, and 0.25 mM phenylmethylsulfonyl fluoride (pH 7.0).

The relaxation solution consisted of 100 mM potassium chloride (KCl), 10 mM imidazole, 1 mM MgCl_2_, 2 mM EGTA, and 4.46 mM Na-ATP.

The glycerol solution for the storage of muscle bundles consisted of 50% (vol/vol) glycerol, 100 mM KCl, 10 mM imidazole, 1 mM MgCl_2_, 2 mM EGTA, and 4.46 mM Na-ATP. The activation solution consisted of 40 mM BES, 10 mM CaCO_3_-EGTA, 6.29 mM MgCl_2_, 6.12 mM Na-ATP, 45.3 mM K-propionate, and 15 mM creatine phosphate. The free Ca^2+^ concentration of pCa was 4.0 (10^−4^ M), where pCa = −log Ca^2+^ concentration for activation.

### 2.5. Functional Study

Single muscle fibres were carefully isolated from muscle bundles after chemical skinning with a skinning solution till 24 h at 4°C. A fibre of between 1 and 2 mm in length was mounted on the needle linked to the force transducer and motor lever arm (Model 403; Aurora Scientific, Aurora, Ontario, Canada) exposed to the solution in the test apparatus. The fibre was stabilized in the relaxing solution with a consistent length of sarcomeres (2.6–2.7 *μ*m, which is considered to be the physiological range of sarcomere length) [18, 19]. The sarcomere length, diameter, and fibre length were measured (ASI software, Aurora Scientific). The diameter was obtained by averaging the length of the diameter seen from the top and the side by using the prism equipped within a microscope (Olympus IX71; Olympus, Tokyo, Japan). The fibre CSA, assumed to be an elliptical shape, was calculated from the diameter and depth. We used a 20% correction when CSA was used for calculating the SF (force/CSA) [20].

**Figure 1:**
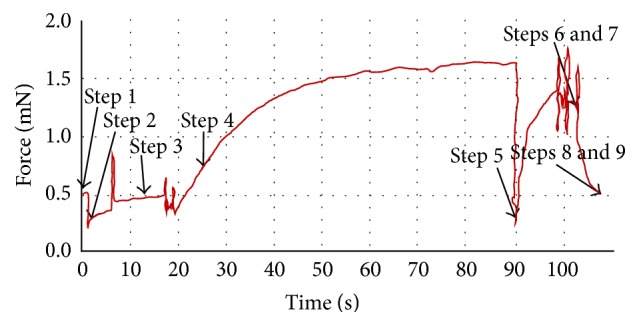
Diagram of the study protocol: 9 steps (see Section 2.5 describing each step in detail). Maximal contractile force (Step  4), maximal shortening velocity (Vo, Step  5) from the slack test, and specific force (maximal contractile force/CSA) were obtained from this protocol.

Maximal velocity (Vo) was measured by using the slack test [21], whereas maximal contractile force was evaluated by using previously described methods [22]. A nine-step experiment was performed for each tested fibre according to the protocol shown in Figure 1: the experiment starts from 0 s (Step  1); the length of a single muscle fibre is shortened to 90% from 1 s (Step  2), and then the length is recovered at 6 s (Step  3); the solution is changed to the activation solution from the relaxation solution at 17 s (Step  4); the fibre length is then slackened by 7, 8, 10, 12, or 13% at 90 s (Step  5) (see Supplement 1 of the Supplementary Material available online at http://dx.doi.org/10.1155/2016/6206959); the solution is changed back to the relaxation solution at 98 s and the original length of the fibre is restored at 102 s (Step  6 and Step  7); finally, the data collection is finished (Step  8) and the protocol is stopped (Step  9). A representative result from this experimentally programmed procedure is shown in Figure 1. The entire experimental procedure was performed at 15.3°C.

### 2.6. Determination of Single Muscle Fibre MHC

**Figure 2:**
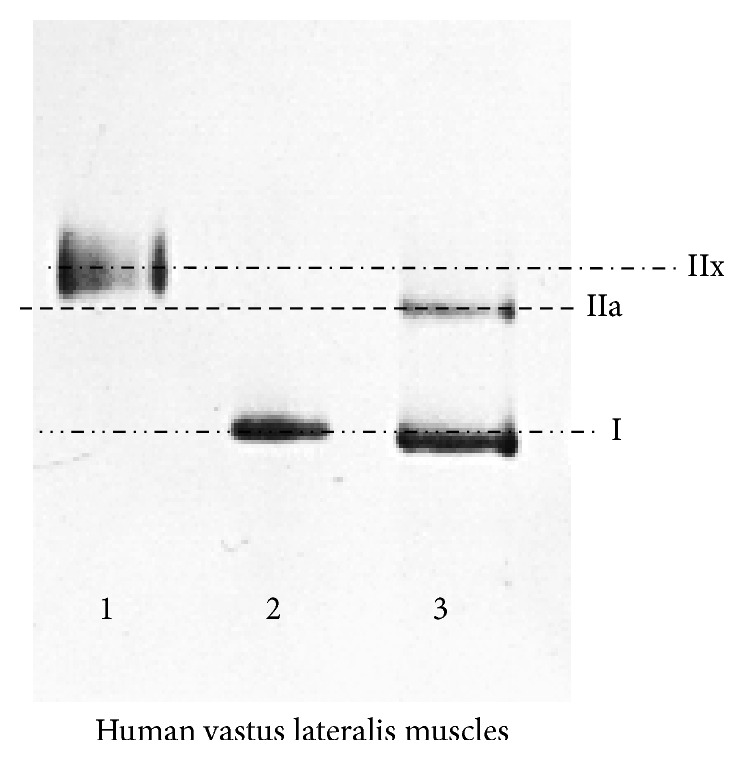
Myosin heavy chain (MHC) separation. Electrophoresis was used for separation of MHC isoforms in the human VL muscle. The top-down method separating MHC isoforms as IIx, IIa, and I is illustrated in the graph. 1 and 3 indicate the MHC standard showing IIx and IIa and I, respectively. 2 indicates a human sample including only MHC I. Using this method, all samples of all subjects were found to be composed of those fibre MHC types.

To determine the isoform of single muscle fibres measured by the method described in Section 2.5, bundles of the fibres were chemically skinned for 24 h in a skinning solution containing 50% (v/v) glycerol at 4°C [23, 24]. A total of 212 fibres were subjected to sodium dodecyl sulphate polyacrylamide gel electrophoresis (SDS-PAGE) for MHC isoform identification after the single muscle fibre test. A gel was used to identify MHC isoforms with a marker as in Figure 2. The MHC composition was determined by using 6% SDS-PAGE. The acrylamide concentration was 4% (w/v) in the stacking gel and 6% in the separating gel, and the gel matrix included 30% glycerol. SDS-PAGE was run at a constant voltage of 90 V for 30 min and 140 V for 5.5 h [22]. A protein homogenate mixture of human muscle MHC I, IIa, and IIx was used as the MHC standard, and the order of migration of each human MHC in the gel is shown according to a previously described method [25] (Figure 2). Gels loaded with single muscle fibres were subjected to silver staining to identify the MHC isoforms of each single muscle fibre, and densitometry was performed by using an analytical software (Bio-1D Light; Vilber Laurat, Marne-la-Vallée, France). IIx, I/IIa, I/IIx, IIa/IIx, and I/IIa/IIx were uniformly treated as I/II hybrids.

### 2.7. Statistical Analysis

**Figure 3:**
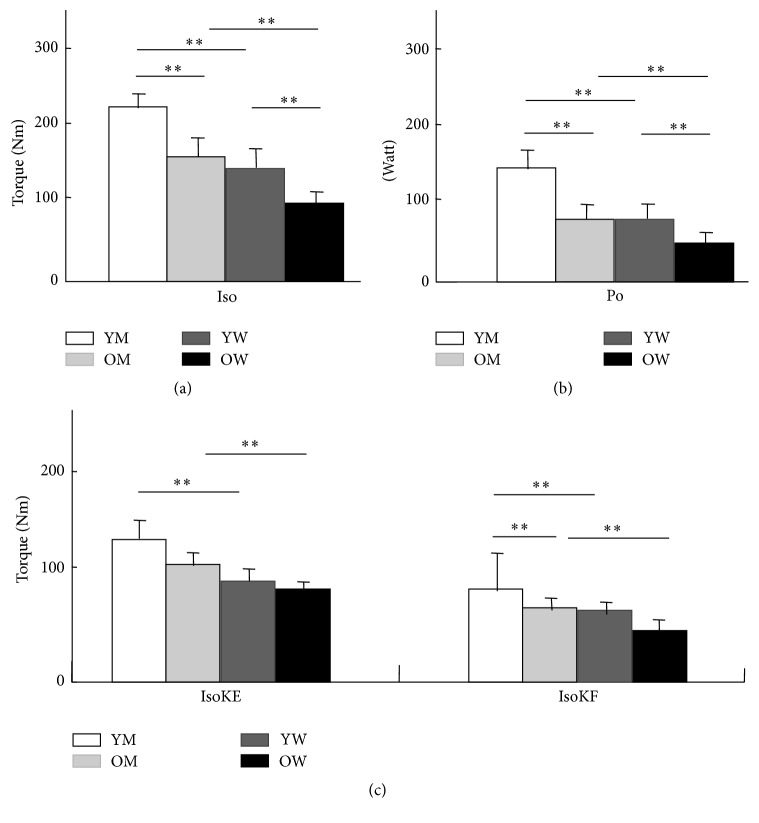
Isometric, power tests, isokinetic peak extension, and isokinetic peak flexion of knee joint. We measured in vivo mechanical properties using BTE before biopsy for the in vitro single muscle fibre study. The voluntary participants for the in vivo study were tested before obtaining isokinetic data of left thigh muscle function. YM, young men; Nm, Newton meter; YM, young men; OM, old men; YW, young women; OW, old women; Iso, isometric test with left thigh; Po, power test; IsoKE, isokinetic test left thigh extension peak torque; IsoKF, isokinetic test left thigh flexion peak torque. The units used in peak values are Nm, newton meter, and watts. ^*∗*^ *p* < 0.05 and ^*∗∗*^ *p* < 0.01 show statistically significant differences. (a) Each Iso value (mean ± standard deviation) of YM, OM, YW, and OW is 221.82 ± 12.64, 156.58 ± 31.49, 143.11 ± 34.33, and 89.29 ± 15.92, respectively. (b) Each Po value of YM, OM, YW, and OW is 141.05 ± 25.72, 83.09 ± 26.18, 82.99 ± 26.02, and 49.86 ± 0.07, respectively. (c) Each IsoKE value of YM, OM, YW, and OW is 125.98 ± 29.90, 94.92 ± 18.30, 79.50 ± 23.00, and 68.76 ± 8.97, respectively, and each IsoKF value of YM, OM, YW, and OW is 90.46 ± 45.63, 49.99 ± 12.00, 41.61 ± 9.29, and 34.03 ± 11.44, respectively. One-way repeated analysis of variance was used. After identifying significant differences among three or more groups, we used the post hoc *t*-test to compare statistical differences between groups.

All data are presented as mean ± standard deviation (SD), except for the analysis with a linear mixed model described as *p* value (Table 2). Each classified value analysed by fibre type from the left VL of subjects (Table 3) and analysed for the power output measurements in this study was compared by using one-way repeated analysis of variance (Figure 3). After identifying significant differences among three or more groups, we used the post hoc *t*-test to compare statistical differences between groups. We also used a linear mixed model for single muscle fibre analysis, which shows the representativeness of each fibre subtype for each subject group [22] (Table 2). SPSS version 18.0 was used for the entire statistical analysis. A value of *p* < 0.05 indicates a statistically significant difference for all analyses, and this level of significance was applied for the same fibres.

## 3. Results

### 3.1. Isokinetic Muscle Performance Study of the Left Thigh

In isometric knee extension, the measured values for YM, OM, YW, and OW were 221.82 ± 12.64, 156.58 ± 31.49, 143.11 ± 34.33, and 89.29 ± 15.92 Nm, respectively (Figure 3(a)). The isokinetic extension and flexion force values of the left thigh were as follows: IsoKE, 125.98 ± 29.90 Nm (YM), 94.92 ± 18.30 Nm (OM), 79.50 ± 23.00 Nm (YW), and 68.76 ± 8.97 Nm (OW); IsoKF, 90.46 ± 45.63 Nm (YM), 49.99 ± 12.00 Nm (OM), 41.61 ± 9.29 Nm (YW), and 34.03 ± 11.44 Nm (OW) (Figure 3(c)). BTE also yielded the following power output values: 141.05 ± 25.72 W (YM), 83.09 ± 26.18 W (OM), 82.99 ± 26.02 W (YW), and 49.86 ± 10.07  W (OW) (Figure 3(b)).

In the comparison between different age groups, we found that there were overall significant differences between YM and OM (*p* < 0.01), except in IsoKE (Figure 3). Significant differences were observed between YW and OW in isometric tests and Po (*p* < 0.01); however, there was no statistical difference between the two groups in IsoKE or IsoKF.

Sex difference, such as between YM and YW and between OM and OW, also significantly affects overall value in Iso, Po, IsoKE, and IsoKF (*p* < 0.01).

### 3.2. Mechanical Properties of Single Muscle Fibres

Concerning the mechanical properties of human VL single muscle fibres, we analysed 212 single muscle fibres and categorized them according to CSA, SF, and Vo (Table 3). The CSA, SF, and Vo of YM and YW showed higher values but were not statistically significantly different from those of OM or OW in MHC type I muscle fibres.

The CSA and Vo of YM were both significantly greater than those of YW in MHC type I fibres (*p* < 0.05) but were not significantly different between OM and OW. MHC type IIa and hybrid fibres showed no statistically significant differences in any of the group comparisons.

### 3.3. Differences in MHC Isoforms among YM, OM, YW, and OW

In the MHC isoforms of single muscle fibres analysis, MHC I was predominantly expressed in all age and sex groups. Other isoforms detected in this study were IIa, IIx, or hybrid (I/IIa/IIx, IIa/IIx, I/IIx, or I/IIa), and we categorized MHC subtypes as I, IIa, and hybrid (I/IIa/IIx, IIa/IIx, I/IIx, I/IIa, or IIx) (Table 3). All groups contain MHC I, IIa, and hybrid subtypes, except for OW, which did not show the MHC IIa subtype. YM consisted of 59% of MHC I and 24% of MHC IIa, whereas OM consisted of MHC I (62%) and MHC IIa (21%). MHC I in YW comprises 86% of whole MHC subtypes. In OW, MHC types I and I/II hybrid are made of 86% and 14%, respectively.

## 4. Discussion

We investigated the mechanical properties of human VL-derived mature single muscle fibres together with the gross functions of the whole thigh muscle in young and elderly men and women. Our results were consistent with those of a previous study showing that the fibre MHC type distribution changes to a more hybrid MHC type with aging [14]. In the comparison between single muscle fibres and whole thigh muscle, sex, rather than age, showed clear differences in mechanical properties. We found that in vitro VL-derived single muscle fibres had no statistically different biomechanical values in similar fibres from the four groups, except for sex differences in the young groups. This is in contrast to the in vivo study, in which identical subjects had remarkable differences in sex and age.

### 4.1. Is Age Difference Also Related to Different Functions of Single Muscle Fibres?

Physical function decreases with aging, and loss of muscle mass and strength is a significant physiological phenomenon. Despite the distinct phenotypic difference caused by aging, the difference of functional properties at the cellular level is not remarkably related to this process [5]. At the single muscle fibre level, it was previously reported that VL-derived type IIa single muscle fibres of YM had a higher value (*p* < 0.01) of contractile velocity than that of OM [3]. Another study reported that this difference is negated when the same type of fibre was compared between the two groups [6]. We also found no significant difference in any parameters of age difference (Tables 2 and 3), although there was a pattern similar to the previous report. The results from our human study at the myocyte level are consistent with those of previous studies [9] and suggest that these differences may arise from different ethnicities, lifestyles, and/or population size.

### 4.2. Does Sex Difference Reflect Functional Differences at the Single Muscle Fibre Level?

The peak strength of muscular contraction differs according to sex. The intrinsic contributor of peak strength loss is derived from neuromuscular function, which is dependent on excitation-contraction coupling and alterations in cross-bridge mechanisms [16, 25–31]. There are different CSA values in each group, and type I in particular has a remarkable pattern compared with the other types (the CSA is greater in men than in women).

This study demonstrated that there is a significant difference only in the CSA and Vo between YM and YW (*p* < 0.05). However, previous studies by Krivickas et al. showed that there was a significant difference in SF for MHC type I and IIa fibres between OM and OW (*p* < 0.05). They also reported a significant difference in Vo between OM and OW for MHC type IIa (*p* < 0.01) [3, 13]. We postulate that the differences of our results from those of previous studies (described above) mainly resulted from the difference in the size of study subjects (916 muscle fibres from 7 YM, 7 YW, 12 OM, and 12 OW, [3]; 307 muscle fibres from 6 OM and 10 OW [13]; 212 muscle fibres from 16 YM, 11 OM, 11 YW, and 7 OW in this study.).

### 4.3. Comparison of Fibre Type Composition

Various mechanical properties are demonstrated by different MHC types of fibres because of the MHC subtype-dependent differences in detachment rates between actin and myosin (actomyosin). This interrelation probably causes the different mechanical properties among different ages and sexes.

Our results do not particularly show that more hybrid MHC types were formed with aging since our cross-sectional study has been limited to finding MHC type changes with aging [32] (Table 3). Frontera et al.'s study in longitudinal research (8.9 yrs) with elderly subjects also supports our results that little changes of MHC type distribution were shown [33]. In particular, the MHC I/IIa/IIx type was found only in the OM and OW groups. MHC I accounted for most of the fibres among all four groups. The most commonly expressed fibre type was MHC IIa in all groups, except for the OW group. This suggests that most of the expressed fibre MHC types within the human VL muscle are typically transformed by aging or mechanical stress to within MHC type I and type IIa (and vice versa); this may also apply to fibre MHC type transformations seen between MHC types I, IIa, and II hybrid [34, 35]. Different signalling pathways are separately susceptible for the responsive expression of specific subtypes of fibres, such as peroxisome proliferator-activated receptor gamma coactivator 1-alpha for MHC type I (protectively induced by inactivity or denervation) and nuclear factor kappa B for MHC type IIx (usually induced by a pathological state and aging) [9].

### 4.4. In Vivo Assessment of Thigh Muscle Power Output and Its Correlation with the In Vitro Study

To comprehensively understand the differences of muscular functionality according to age and sex, we added an in vivo study in which we assessed the quadriceps muscle force. Generally, whole thigh muscles show patterns in which age- and sex-related changes are reflected by differences in muscle force output performance. We showed that different sexes, rather than different ages, have a clearer pattern of different mechanical properties at both the single muscle fibre and whole thigh muscle levels. Furthermore, the in vivo mechanical properties of different groups were remarkably different from that of single muscle fibre function.

These factors may also be positively changed by beneficial interventions. As optimal alternatives, resistant training usually transforms the mechanical properties of slow myofibres [27]. The aerobic and eccentric contraction induced by training possibly improves the mechanical properties of fast fibres [36–38]. Fast fibres produce five to six times the power output of slow fibres, and this is a better choice for alternative therapeutic exercises to ameliorate the effects of aging [39].

One of the limitations of this in vivo study was that we could not obtain precise mechanical data from only the accurate function of the VL to link to those of the in vitro VL-derived single muscle fibre analysis. Mechanisms controlling the properties of the human skeletal muscle at a molecular level may serve as a better model to describe the overall characteristics of the muscle for designing positive interventions in future studies.

In conclusion, we used powerful methods to measure in vitro muscular function that simulates the physiological state at a single muscle level. Human VL-derived single muscle fibres from subjects of different ages and sexes were classified according to fibre MHC types. Human VL muscle overwhelmingly consists of MHC I in all groups. There were significant differences in the CSA and Vo between YM and YW (*p* < 0.05). Our results showed that age and sex differences seemingly reflect mechanical properties at both the single muscle fibre level and whole thigh muscle level; however, the sex differences were more remarkable at both levels. Furthermore, we found that various functional properties among different groups were more prominent in the whole thigh muscle than in single muscle fibres. These data provide a greater understanding of the characteristics of the human muscle and should contribute to the development of interventions for competitive sports, treatments of muscle-related diseases, prevention of sarcopenia, and advancement of muscle-related research.

## Supplementary Material

In this video, a human single muscle fiber mounted on in-vitro apparatus was contracted in activation solution and then became relax again in relaxation solution.

## Figures and Tables

**Table 1:** General characteristics of subjects.

	Ages (year)	Height (cm)	Weight (kg)
YM (*n* = 16)	29.25 ± 4.65	176.84 ± 2.52	75.28 ± 9.15
OM (*n* = 11)	71.45 ± 2.94	164.10 ± 5.18	68.24 ± 6.51
YW (*n* = 11)	29.64 ± 4.88	160.35 ± 4.57	56.79 ± 10.64
OW (*n* = 7)	67.29 ± 1.70	155.69 ± 6.10	58.01 ± 9.71

Values are presented as means ± standard deviation. YM = young men, OM = old men, YW = young women, OW = old women.

**Table 2:** *p* values of multilevel analysis for correlated single muscle fiber with each subject group.

Fiber type	Classification	CSA	SF (mN/mm^2^)	Vo (FL/s)	F (mN)
I	Age	0.774	0.569	0.528	0.612
	Gender	0.179	0.254	0.059	0.168
IIa	Age	0.101	0.938	0.683	0.146
	Gender	0.797	0.759	0.221	0.624
I/II hybrid	Age	0.753	0.916	0.677	0.928
	Gender	0.490	0.702	0.166	0.453

One of the multilevel analyses, linear mixed model, was used for showing the representativeness of each fiber subtype for each subject group.

Total fiber number = 212. Values are estimated as means ± SD. CSA = cross-sectional area, SF = specific force, Vo = maximum shortening velocity,

F = maximal contractile force.

**Table 3:** Characteristics of human vastus lateralis single muscle fiber.

Classification	Fiber type	CSA (*μ*m^2^)	SF (mN/mm^2^)	Vo (FL/s)	F (mN)
YM	I (*n* = 65)	4679.69 ± 1143.48^*∗*^	125.64 ± 73.04	1.69 ± 1.19^*∗*^	0.50 ± 0.41
	IIa (*n* = 27)	4779.22 ± 844.51	131.86 ± 67.16	2.25 ± 1.60	0.52 ± 0.37
	I/II hybrid (*n* = 19)	5202.21 ± 731.65	137.70 ± 66.19	2.77 ± 1.46	0.59 ± 0.35
OM	I (*n* = 21)	4503.95 ± 1769.54	123.34 ± 38.79	1.18 ± 0.93	0.45 ± 0.25
	IIa (*n* = 7)	3573.14 ± 1943.94	129.28 ± 114.56	1.84 ± 0.67	0.30 ± 0.20
	I/II hybrid (*n* = 6)	4962.83 ± 2574.96	138.64 ± 68.53	1.95 ± 1.12	0.59 ± 0.42
YW	I (*n* = 25)	4108.72 ± 103.17^*∗*^	116.99 ± 35.40	0.85 ± 0.35^*∗*^	0.39 ± 0.16
	IIa (*n* = 4)	4436.50 ± 917.72	118.92 ± 75.58	0.99 ± 0.21	0.43 ± 0.28
	I/II hybrid (*n* = 2)	4001.50 ± 901.56	122.87 ± 32.76	0.62 ± 0.08	0.38 ± 0.01
OW	I (*n* = 31)	4050.13 ± 1072.37	104.53 ± 41.87	0.97 ± 0.42	0.34 ± 0.19
	IIa (*n* = 0)	ND	N/D	N/D	N/D
	I/II hybrid (*n* = 5)	4656.80 ± 429.57	129.38 ± 70.47	1.99 ± 1.32	0.50 ± 0.30

One-way repeated analysis of variance followed by the post hoc *t*-test was used for statistical significance. Values are presented as means ± standard deviation. YM = young men, OM = old men, YW = young women, OW = old women, CSA = cross-sectional area, SF = specific force, Vo = maximum shortening velocity, F = maximal contractile force, ND = not detected. ^*∗*^ *p* < 0.05: statistical significances between YM type I and YW type I (*p* = 0.021 for CSA and *p* = 0.016 for CV).

**Table 4:** Dates of degraded quality in single muscle fiber mechanics.

Classification	Fiber type	CSA (*μ*m^2^)	SF (mN/mm^2^)	Vo (FL/s)	F (mN)	Stored days
YM	I (*n* = 7),	4655.93 ± 807.40	36.02 ± 8.65	2.34 ± 1.04	0.13 ± 0.04	16.67 ± 19.07
	IIa (*n* = 6),					
	I/II hybrid (*n* = 2)					

OM	I (*n* = 2),	4010.57 ± 2716.34	45.02 ± 18.10	2.17 ± 1.57	0.15 ± 0.14	28.57 ± 18.54
	IIa (*n* = 1),					
	I/II hybrid (*n* = 3)					

YW	I (*n* = 11)	3582.36 ± 871.25	40.82 ± 15.12	0.83 ± 0.22	0.12 ± 0.05	21.27 ± 22.35

OW	I (*n* = 7),	3697.38 ± 654.92	45.01 ± 17.33	0.80 ± 0.28	0.13 ± 0.05	40.63 ± 23.32
	I/II hybrid (*n* = 1)					

Values are presented as means ± standard deviation. YM = young men, OM = old men, YW = young women, OW = old women, CSA = cross-sectional area, SF = specific force, Vo = maximum shortening velocity, F = maximal contractile force.

## References

[1] Lexell J., Taylor C. C., Sjöström M. (1988). What is the cause of the ageing atrophy? Total number, size and proportion of different fiber types studied in whole vastus lateralis muscle from 15- to 83-year-old men. *Journal of the Neurological Sciences*.

[2] Short K. R., Vittone J. L., Bigelow M. L. (2005). Changes in myosin heavy chain mRNA and protein expression in human skeletal muscle with age and endurance exercise training. *Journal of Applied Physiology*.

[3] Krivickas L. S., Suh D., Wilkins J., Hughes V. A., Roubenoff R., Frontera W. R. (2001). Age- and gender-related differences in maximum shortening velocity of skeletal muscle fibers. *American Journal of Physical Medicine & Rehabilitation*.

[4] Korhonen M. T., Cristea A., Alén M. (2006). Aging, muscle fiber type, and contractile function in sprint-trained athletes. *Journal of Applied Physiology*.

[5] Canepari M., Pellegrino M. A., D'Antona G., Bottinelli R. (2010). Single muscle fiber properties in aging and disuse. *Scandinavian Journal of Medicine and Science in Sports*.

[6] Trappe S., Gallagher P., Harber M., Carrithers J., Fluckey J., Trappe T. (2003). Single muscle fibre contractile properties in young and old men and women. *The Journal of Physiology*.

[7] Janssen I., Heymsfield S. B., Wang Z., Ross R. (2000). Skeletal muscle mass and distribution in 468 men and women aged 18–88 yr. *Journal of Applied Physiology*.

[8] Lindle R. S., Metter E. J., Lynch N. A. (1997). Age and gender comparisons of muscle strength in 654 women and men aged 20–93 yr. *Journal of Applied Physiology*.

[9] Miljkovic N., Lim J.-Y., Miljkovic I., Frontera W. R. (2015). Aging of skeletal muscle fibers. *Annals of Rehabilitation Medicine*.

[10] Kostka T. (2005). Quadriceps maximal power and optimal shortening velocity in 335 men aged 23–88 years. *European Journal of Applied Physiology*.

[11] Kostka T., Bonnefoy M., Arsac L. M., Berthouze S. E., Belli A., Lacour J.-R. (1997). Habitual physical activity and peak anaerobic power in elderly women. *European Journal of Applied Physiology and Occupational Physiology*.

[12] Bonnefoy M., Kostka T., Arsac L. M., Berthouze S. E., Lacour J.-R. (1998). Peak anaerobic power in elderly men. *European Journal of Applied Physiology and Occupational Physiology*.

[13] Krivickas L. S., Fielding R. A., Murray A. (2006). Sex differences in single muscle fiber power in older adults. *Medicine and Science in Sports and Exercise*.

[14] Snow L. M., McLoon L. K., Thompson L. V. (2005). Adult and developmental myosin heavy chain isoforms in soleus muscle of aging Fischer Brown Norway rat. *Anatomical Record—Part A Discoveries in Molecular, Cellular, and Evolutionary Biology*.

[15] Frontera W. R., Hughes V. A., Krivickas L. S., Kim S.-K., Foldvari M., Roubenoff R. (2003). Strength training in older women: early and late changes in whole muscle and single cells. *Muscle & Nerve*.

[16] Frontera W. R., Suh D., Krivickas L. S., Hughes V. A., Goldstein R., Roubenoff R. (2000). Skeletal muscle fiber quality in older men and women. *American Journal of Physiology—Cell Physiology*.

[17] Trombetti A., Reid K. F., Hars M. (2016). Age-associated declines in muscle mass, strength, power, and physical performance: impact on fear of falling and quality of life. *Osteoporosis International*.

[18] Narici M., Franchi M., Maganaris C. (2016). Muscle structural assembly and functional consequences. *Journal of Experimental Biology*.

[19] Brown S. H. M., Gerling M. E. (2012). Importance of sarcomere length when determining muscle physiological cross-sectional area: a spine example. *Proceedings of the Institution of Mechanical Engineers, Part H: Journal of Engineering in Medicine*.

[20] Godt R. E., Maughan D. W. (1977). Swelling of skinned muscle fibers of the frog. Experimental observations. *Biophysical Journal*.

[21] Edman K. A. P. (1979). The velocity of unloaded shortening and its relation to sarcomere length and isometric force in vertebrate muscle fibres. *The Journal of Physiology*.

[22] Frontera W. R., Choi H., Krishnan G., Krivickas L. S., Sabharwal S., Teng Y. D. (2006). Single muscle fiber size and contractility after spinal cord injury in rats. *Muscle and Nerve*.

[23] Wang K., McClure J., Tu A. (1979). Titin: major myofibrillar components of striated muscle. *Proceedings of the National Academy of Sciences of the United States of America*.

[24] Pachter B. R., Eberstein A. (1991). Nerve sprouting and endplate growth induced in normal muscle by contralateral partial denervation of rat plantaris. *Brain Research*.

[25] Widrick J. J., Maddalozzo G. F., Lewis D. (2003). Morphological and functional characteristics of skeletal muscle fibers from hormone-replaced and nonreplaced postmenopausal women. *Journals of Gerontology, Series A: Biological Sciences and Medical Sciences*.

[26] Larsson L., Grimby G., Karlsson J. (1979). Muscle strength and speed of movement in relation to age and muscle morphology. *Journal of Applied Physiology Respiratory Environmental and Exercise Physiology*.

[27] Klitgaard H., Mantoni M., Schiaffino S. (1990). Function, morphology and protein expression of ageing skeletal muscle: a cross-sectional study of elderly men with different training backgrounds. *Acta Physiologica Scandinavica*.

[28] Brooks S. V., Faulkner J. A. (1988). Contractile properties of skeletal muscles from young, adult and aged mice. *Journal of Physiology*.

[29] Thompson L. V., Brown M. (1999). Age-related changes in contractile properties of single skeletal fibers from the soleus muscle. *Journal of Applied Physiology*.

[30] Delbono O., O'Rourke K. S., Ettinger W. H. (1995). Excitation-calcium release uncoupling in aged single human skeletal muscle fibers. *The Journal of Membrane Biology*.

[31] Larsson L., Li X., Frontera W. R. (1997). Effects of aging on shortening velocity and myosin isoform composition in single human skeletal muscle cells. *American Journal of Physiology—Cell Physiology*.

[32] Wang Y., Pessin J. E. (2013). Mechanisms for fiber-type specificity of skeletal muscle atrophy. *Current Opinion in Clinical Nutrition and Metabolic Care*.

[33] Frontera W. R., Reid K. F., Phillips E. M. (2008). Muscle fiber size and function in elderly humans: a longitudinal study. *Journal of Applied Physiology*.

[34] Jee H., Sakurai T., Lim J., Hatta H. (2014). Changes in *α*B-crystallin, tubulin, and MHC isoforms by hindlimb unloading show different expression patterns in various hindlimb muscles. *Journal of Exercise Nutrition and Biochemistry*.

[35] Malisoux L., Francaux M., Theisen D. (2007). What do single-fiber studies tell us about exercise training?. *Medicine and Science in Sports and Exercise*.

[36] Drexel H., Saely C. H., Langer P. (2008). Metabolic and anti-inflammatory benefits of eccentric endurance exercise—a pilot study. *European Journal of Clinical Investigation*.

[37] Zeppetzauer M., Drexel H., Vonbank A., Rein P., Aczel S., Saely C. H. (2013). Eccentric endurance exercise economically improves metabolic and inflammatory risk factors. *European Journal of Preventive Cardiology*.

[38] Proctor D. N., Sinning W. E., Walro J. M., Sieck G. C., Lemon P. W. R. (1995). Oxidative capacity of human muscle fiber types: effects of age and training status. *Journal of Applied Physiology*.

[39] Harber M. P., Konopka A. R., Douglass M. D. (2009). Aerobic exercise training improves whole muscle and single myofiber size and function in older women. *American Journal of Physiology—Regulatory Integrative and Comparative Physiology*.

